# A Nomogram Based on Preoperative Inflammatory Indices and ICG-R15 for Prediction of Liver Failure After Hepatectomy in HCC Patients

**DOI:** 10.3389/fonc.2021.667496

**Published:** 2021-07-02

**Authors:** Tongdi Fang, Guo Long, Dong Wang, Xudong Liu, Liang Xiao, Xingyu Mi, Wenxin Su, Liuying Zhou, Ledu Zhou

**Affiliations:** ^1^ Department of General Surgery, The Xiangya Hospital of Central South University, Changsha, China; ^2^ Department of Liver Disease Center, The Affiliated Hospital of Qingdao University, Qingdao, China; ^3^ Department of Orthopedics Surgery, The Second Xiangya Hospital of Central South University, Changsha, China; ^4^ Medical Record Management and Information Statistics Center, The Xiangya Hospital of Central South University, Changsha, China

**Keywords:** ICG-R15, APRI, nomogram, hepatocellular carcinoma, post-hepatectomy liver failure

## Abstract

**Objective:**

To establish a nomogram based on inflammatory indices and ICG-R15 for predicting post-hepatectomy liver failure (PHLF) among patients with resectable hepatocellular carcinoma (HCC).

**Methods:**

A retrospective cohort of 407 patients with HCC hospitalized at Xiangya Hospital of Central South University between January 2015 and December 2020, and 81 patients with HCC hospitalized at the Second Xiangya Hospital of Central South University between January 2019 and January 2020 were included in the study. Totally 488 HCC patients were divided into the training cohort (n=378) and the validation cohort (n=110) by random sampling. Univariate and multivariate analysis was performed to identify the independent risk factors. Through combining these independent risk factors, a nomogram was established for the prediction of PHLF. The accuracy of the nomogram was evaluated and compared with traditional models, like CP score (Child-Pugh), MELD score (Model of End-Stage Liver Disease), and ALBI score (albumin-bilirubin) by using receiver operating characteristic (ROC) curve, calibration curve, and decision curve analysis (DCA).

**Results:**

Cirrhosis (OR=2.203, 95%CI:1.070-3.824, P=0.030), prothrombin time (PT) (OR=1.886, 95%CI: 1.107-3.211, P=0.020), tumor size (OR=1.107, 95%CI: 1.022-1.200, P=0.013), ICG-R15% (OR=1.141, 95%CI: 1.070-1.216, P<0.001), blood loss (OR=2.415, 95%CI: 1.306-4.468, P=0.005) and AST-to-platelet ratio index (APRI) (OR=4.652, 95%CI: 1.432-15.112, P=0.011) were independent risk factors of PHLF. Nomogram was built with well-fitted calibration curves on the of these 6 factors. Comparing with CP score (C-index=0.582, 95%CI, 0.523-0.640), ALBI score (C-index=0.670, 95%CI, 0.615-0.725) and MELD score (C-ibasedndex=0.661, 95%CI, 0.606-0.716), the nomogram showed a better predictive value, with a C-index of 0.845 (95%CI, 0.806-0.884). The results were consistent in the validation cohort. DCA confirmed the conclusion as well.

**Conclusion:**

A novel nomogram was established to predict PHLF in HCC patients. The nomogram showed a strong predictive efficiency and would be a convenient tool for us to facilitate clinical decisions.

## Introduction

With the rapid rise in its prevalence, hepatocellular carcinoma (HCC) has become the sixth most aggressive malignant tumor and the second-leading cause of cancer-related deaths worldwide (1). Among all therapeutic strategies, surgical resection remain the mainstay of the curative approach for HCC nowadays (2). Even though surgical technique and perioperative care have significantly improved over the past few years, post-hepatectomy liver failure (PHLF) is still the primary driver of morbidity and mortality after hepatectomy in HCC patients (3, 4). In patients with low liver regeneration capability and reduced function reservation of remnant liver tissue following hepatectomy, PHLF occurs most frequently. Therefore, it is critically important to predict the risk of PHLF, which is essential for surgeons to choose individualized treatment.

To accurately predict PHLF, several articles relating to PHLF have been published (5–8). Although these efforts on the preoperative prediction of PHLF have been made, an effective prediction model is still lacking (9). For many years, the clinical scoring systems, such as CP score (Child-Pugh) (10, 11) and MELD score (Model of End-Stage Liver Disease) (12), are widely used for preoperative assessment of liver function. The Child-Pugh score system has some drawbacks and limitations because of its two subjective clinical variables-ascites and hepatic encephalopathy (11). Similarly, the MELD score is not optimal for the prediction of PHLF. Recent research indicated that a new evidence-based model, called the albumin-bilirubin (ALBI) score (13). has been developed to assess liver function reserve. And it has been proven to be superior in estimating PHLF and survival of HCC patients undergoing liver resection (14, 15). Indocyanine green (ICG) (16), a nontoxic, infrared, and photosensitive dye, can be combined with albumin and beta lipoprotein. As a quantitative test to assess hepatic blood flow and liver function, the ICG clearance test at 15 minutes (ICG-R15) is now commonly used to evaluate reserved liver function in surgical patients. Moreover, ICG-R15 has been proven to be a reliable predictor of PHLF recently (17, 18).

Serum inflammatory indices are a reflection of the systemic inflammatory, which plays a significant role in the pathogenesis and progression of liver cirrhosis (19). Recently, a study has demonstrated that chronic inflammation can increase the operative risk of liver resection (20). However, the exact relationship between Serum inflammatory indices and PHLF is not very clear. And whether the combination of inflammatory indices and ICG-R15 could add more benefit in predicting PHLF is worth exploring.

Therefore, this study aims to investigate the possibility of inflammatory markers in predicting PHLF. Moreover, we develop a nomogram based on ICG-R15 and inflammatory markers, and compare its predictive value with traditional models, such as CP score, MELD score and ALBI score, in HCC patients undergoing hepatectomy.

## Patients and Methods

### Patients

We retrospectively collected the data from 488 HCC patients who underwent partial hepatectomy from the Xiangya Hospital of Central South University and the Second Xiangya Hospital of Central South University in China. By random sampling, 378 patients were selected as training cohorts while another 110 patients were chosen as the validation cohorts. The study was approved by the Ethics Committee of the Xiangya Hospital of Central South University and the Second Xiangya Hospital of Central South University in compliance with the Declaration of Helsinki. Written informed consent was obtained from all patients for use of their data in this study. Barcelona Clinic Liver Cancer (BCLC) criteria were applied to select HCC patients for hepatectomy in the paper.

The inclusion criteria were as follows: 1) diagnosis of HCC confirmed by histology; 2) no anticancer treatments before hepatectomy, including transarterial chemoembolization, radiofrequency ablation, and others; 3) no simultaneous malignancies; 4) no preoperative obstructive jaundice; and 5) no preoperative cardiopulmonary, renal dysfunction or severe encephalopathy.

The exclusion criteria were as follows: 1) Patients with recurrent tumors before hepatectomy; 2) no liver function and coagulation function data on or after postoperative day 5.

### Indocyanine Green Test

Prior to surgery, a dose of 50mg ICG (Yichuang Pharmaceutical Co. Ltd., China) dissolved in 10ml of sterile water was injected through a peripheral vein based on the bodyweight of patients (0.5mg/kg). The 15-min retention rate of ICG (ICG-R15) was measured at 15 min after injection using a pulse spectrophotometer (DDG-3300K, Japan). Results were expressed as the percentage of ICG-R15 after injection.

### Clinicopathologic Variables

Patients’ demographic variables were collected including age, gender, history of diabetes, hypertension, hepatitis B based on discharge diagnosis. The number of tumor nodules, tumor size(major nodule diameter), cirrhosis and ascites were included in patients’ imaging data based on contrast-enhanced MRI, contrast-enhanced CT and ultrasound. The following data were recorded based on intraoperative situation: time of operation, blood loss. Preoperative serum examination included serum α-fetoprotein level (AFP), ICG-R15, creatinine (Cr), albumin (ALB), total bilirubin (TBIL), direct bilirubin (DBIL), alanine transaminase (ALT), aspartate transaminase (AST), prothrombin time (PT), international normalized ratio (INR), neutrophil, lymphocyte, monocyte, platelet, hemoglobin, neutrophil-to-lymphocyte ratio (NLR), platelet-to-lymphocyte ratio (PLR), AST-to-platelet ratio index (APRI), lymphocyte-to-monocyte ratio (LMR), and AST-to-neutrophil ratio index (ANRI). ALBI score = 0.66 × lg (TBIL, μmol/L) – 0.085 ×(ALB, g/L). The MELD score = 11.2 × ln (INR) + 9.57 × ln (Cr, mg/dL) + 3.78 × ln (TBIL, mg/dL) + 6.43. APRI = [AST level (/ULN)/Platelet counts (109/L)] × 100. NLR was determined by the neutrophils count divided by lymphocytes count. PLR was measured by the platelet count divided by lymphocytes count. LMR was calculated by the lymphocytes count divided by monocytes count. ANRI was calculated by the AST divided by neutrophils count. All preoperative assessments including ICG, blood routine test, liver function test, AFP, and imaging material was arranged on the 1st day after administration (within 1 week before surgery).

### Definitions of PHLF

There are various definitions of PHLF that have been used. For example, in the study of Eguschi et al (21), PHLF was diagnosed when three results were present in the patient: (1) hepatic encephalopathy, (2) progressive hyperbilirubinemia, (3) reduced hepaplastin test. The “50-50 criteria” is another definition of PHLF proposed by Balzan (22). But it has some limitations due to its high specificity (97.7%) and low sensitivity (69.6%).

It is in 2010 that a consensus about PHLF was reached. PHLF was defined as a postoperatively acquired deterioration in the ability of the liver to maintain its synthetic, excretory, and detoxifying functions, which are characterized by an increased INR and concomitant hyperbilirubinemia on or after postoperative day 5 by the International Study Group of Liver Surgery (ISGLS) (3). So the diagnosis of PHLF in our study is based on the definition of PHLF proposed by ISGLS.

### Statistical Analysis

Continuous variables were expressed as mean ± SD and compared using the Student’s *t*-test or Mann–Whitney *U* test. Categorical variables were shown as frequency and compared using either chi-square test or Fisher exact test. Factors whose P values were less than 0.05 in the univariate analysis were subjected to multivariate logistic regression analysis to identify the independent predictors of PHLF. According to the independent PHLF predictors, a nomogram was plotted by using the *rms* package of R (version 4.0.3). ROC curve analysis was used for comparison between our nomogram and other models based on the concordance index (C index). A calibration plot with 1000 bootstrap samples was employed to measure the accuracy of the nomogram. The decision curve analysis (DCA) was conducted to estimate the clinical usefulness of the nomogram through quantifying net benefits at different threshold probabilities. SPSS 26.0 (SPSS Inc, Chicago, IL, USA) and R 4.0.3 software (Institute for Statistics and Mathematics) were performed in our analysis. P< 0.05 was considered statistically significant.

## Results

### Clinicopathologic Characteristics of Patients

During the study period, 488 patients who met the inclusion criteria were enrolled, including 417 (85.45%) males and 71 (14.55%) females, and divided into the training cohort and validation cohort. The mean age of the 488 patients was 53.08 ± 11.68 (range from 18 to 83) years. The majority of patients (85.24%) were infected with hepatitis B virus (HBV) and cirrhosis was observed in 346 (70.9%) patients. The mean tumor size was (6.53 ± 4.29) cm and 87 (17.83%) patients had multiple tumors. The mean ICG-R15 and APRI were 7.59 ± 7.33 and 0.91 ± 0.74, respectively. PHLF occurred in 42.8% of patients (n =209).

In the training cohort, 378 patients were enrolled and PHLF occurred in 163 patients. For the validation cohort, 110 patients were studied and PHLF occurred in 46 patients. The clinicopathologic characteristics of the patients are listed in **Table 1**. The baseline clinicopathologic data were comparable between the training and validation cohorts.

**Table 1:** Characteristics of patients in training cohort and validation cohort.

Characteristics	Training (n=378)	Validation (n=110)	P value
Age, years	53.29±11.74	52.36±11.50	0.463
Gender		
Male	320	97	0.356
Female	58	13	
Diabetes		
Yes	41	19	0.071
No	337	91	
Hypertension		
Yes	104	27	0.536
No	274	83	
HBsAg		
Positive	321	95	0.707
Negative	57	15	
Cirrhosis		
Yes	261	85	0.095
No	117	25	
Neutrophil, 10^9^/L	3.31±1.32	3.01±1.19	0.031
Lymphocyte, 10^9^/L	1.42±0.49	1.49±0.63	0.234
Monocyte, 10^9^/L	0.46±0.20	0.44±0.18	0.418
Platelet, 10^9^/L	158.80±80.65	160.32±77.75	0.861
HB, g/L	139.00±18.38	142.07±17.17	0.118
TBil, μmol/L	13.24±6.04	13.24±5.15	0.994
DBil, μmol/L	6.00±3.20	6.04±2.48	0.926
Alb, g/L	39.90±4.53	39.74±4.35	0.737
ALT, U/L	40.98±37.32	37.94±22.16	0.415
AST, U/L	49.30±38.50	44.56±32.32	0.240
PT, s	13.47±1.39	13.57±1.27	0.485
INR	1.08±0.11	1.09±0.10	0.335
Cre, μmol/L	84.12±19.69	83.98±17.16	0.949
AFP, ng/ml			
≥400	148	39	0.482
<400	230	71	
Tumor size, cm	6.66±4.31	6.06±4.17	0.194
Tumor number			
Solitary	307	94	0.307
Multiple	71	16	
ICG-R15 (%)	7.38±6.40	8.33±9.90	0.234
Blood loss, ml			
≥400	226	63	0.637
<400	152	47	
Operation time,min	206.10±68.30	204.47±61.04	0.822
NLR	2.57±1.38	2.23±1.06	0.006
PLR	119.44±66.87	114.99±57.25	0.527
LMR	3.45±1.37	3.75±1.65	0.055
ANRI	16.66±13.14	17.08±14.25	0.771
APRI	0.92±0.74	0.88±0.75	0.617

Categorical variables are expressed as frequency. Continuous variables are expressed as mean (standard deviation).

ICG-R15, indocyanine green retention rate at 15 min; AFP, α-fetoprotein level; HBsAg, hepatitis be antigen; HB, hemoglobin; ALB, albumin; TBIL, total bilirubin; DBIL, direct bilirubin; ALT, alanine transaminase; AST, aspartate transaminase; PT, prothrombin time; INR, international normalized ratio; NLR, neutrophil-to-lymphocyte ratio; PLR, platelet-to-lymphocyte ratio; LMR, lymphocyte-to-monocyte ratio; APRI, AST-to-platelet ratio index; ANRI, AST-to-neutrophil ratio index.

### Univariate and Multivariate Analysis of Factors of PHLF

In the training cohort, the univariate analysis suggested that gender (P=0.023), cirrhosis (P<0.001), lymphocyte (P<0.001), platelet (P<0.001), TBil (P<0.001), DBil (P<0.001), Alb (P<0.001), ALT (P<0.001), AST (P<0.001), PT (P<0.001), INR (P<0.001), tumor size (P=0.002), ICG-15R% (P<0.001), blood loss (P<0.001), operation time (P=0.006), NLR (P=0.025), LMR (P<0.001), ANRI (P<0.001) and APRI (P<0.001) were potential risk factors of PHLF. Then, all these potential risk factors were accepted into the multivariate logistic analysis. Only cirrhosis (P=0.030), PT (P=0.020), tumor size (P=0.013), ICG-R15% (P<0.001), blood loss (P=0.005) and APRI (P=0.011) were independent risk factors of PHLF (**Table 2**).

**Table 2:** Univariable And Multivariable Analyses for preoperative and intraoperative variables of PHLF according to ISGLS criteria in the training cohort.

Variables	Univariable logistic regression		Multivariable logistic regression	
	OR (95%CI)	P value	OR (95%CI)	P value
Age, years	1.018 (1.000-1.036)	0.050		
Gender, (Female vs Male)	0.494 (0.269-0.906)	0.023		
Diabetes, (Yes vs No)	1.158 (0.604-2.219)	0.659		
Hypertension, (Yes vs No)	1.468 (0.933-2.312)	0.097		
HBsAg, (Yes vs No)	1.360 (0.760-2.433)	0.300		
Cirrhosis, (Yes vs No)	2.839 (1.762-4.574)	<0.001	2.203 (1.070-3.824)	0.030
Neutrophil, 10^9^/L	0.921 (0.787-1.078)	0.305		
Lymphocyte, 10^9^/L	0.291 (0.179-0.474)	<0.001		
Monocyte, 10^9^/L	1.596 (0.561-4.537)	0.381		
Platelet, 10^9^/L	0.995 (0.992-0.998)	<0.001		
HB, g/L	1.003 (0.992-1.014)	0.629		
TBil, μmol/L	1.075 (1.035-1.117)	<0.001		
DBil, μmol/L	1.192 (1.097-1.296)	<0.001		
Alb, g/L	0.909 (0.867-0.954)	<0.001		
ALT, U/L	1.012 (1.004-1.020)	0.002		
AST, U/L	1.018 (1.010 1.025)	<0.001		
PT, s	1.552 (1.299-1.853)	<0.001	1.886 (1.107-3.211)	0.020
INR	1.761 (1.401-2.213)	<0.001		
Cre, μmol/L	1.004 (0.994-1.014)	0.460		
AFP, (≥400 vs<400 ng/ml)	1.208 (0.796-1.832)	0.374		
Tumor size, cm	1.081 (1.030-1.135)	0.002	1.107 (1.022-1.200)	0.013
Tumor number, (≥2 vs<2 )	1.677 (0.998-2.817)	0.051		
ICG-R15 (%)	1.169 (1.115-1.226)	<0.001	1.141 (1.070-1.216)	<0.001
Blood loss, (≥400 vs<400 ml)	2.870 (1.850-4.452)	<0.001	2.415 (1.306-4.468)	0.005
Operation time, min	1.004 (1.001-1.007)	0.006		
NLR	1.194 (1.022-1.395)	0.025		
PLR	0.999 (0.996-1.002)	0.594		
LMR	0.725 (0.614-0.856)	<0.001		
ANRI	1.064 (1.041-1.089)	<0.001		
APRI	7.176 (4.212-12.226)	<0.001	4.652 (1.432-15.112)	0.011

ICG-R15, indocyanine green retention rate at 15 min; AFP, α-fetoprotein level; HBsAg, hepatitis be antigen; HB, hemoglobin; ALB, albumin; TBIL, total bilirubin; DBIL, direct bilirubin; ALT, alanine transaminase; AST, aspartate transaminase; PT, prothrombin time; INR, international normalized ratio; NLR, neutrophil-to-lymphocyte ratio; PLR, platelet-to-lymphocyte ratio; LMR, lymphocyte-to-monocyte ratio; APRI, AST-to-platelet ratio index; ANRI, AST-to-neutrophil ratio index.

### Nomogram for Post-Hepatectomy Liver Failure

**Figure 1:**
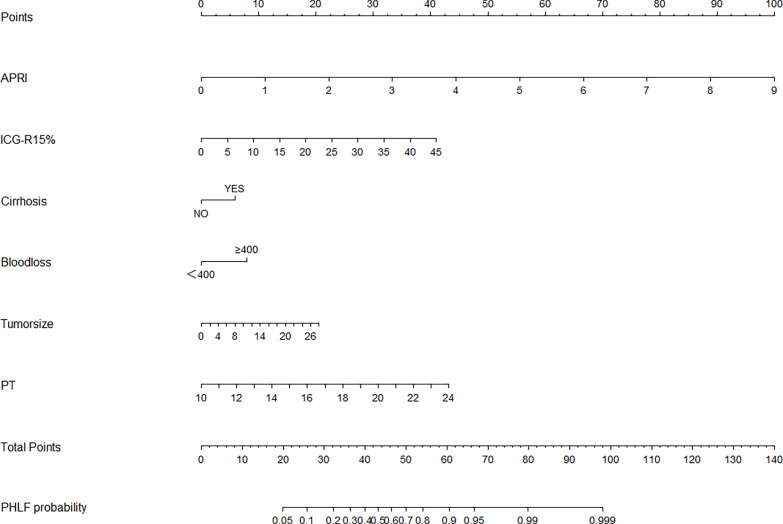
The nomogram was developed in the training cohort and incorporated the AST-to-platelet ratio index (APRI), ICG-R15, tumor size, blood loss, cirrhosis, and prothrombin time (PT). To use the nomogram, an individual patient’s value is located on each variable axis, and a line is drawn upward to determine the number of points received for each variable value. The sum of these points is located on the total points axis, and a line is drawn downward to the likelihood of PHLF.

**Figure 2:**
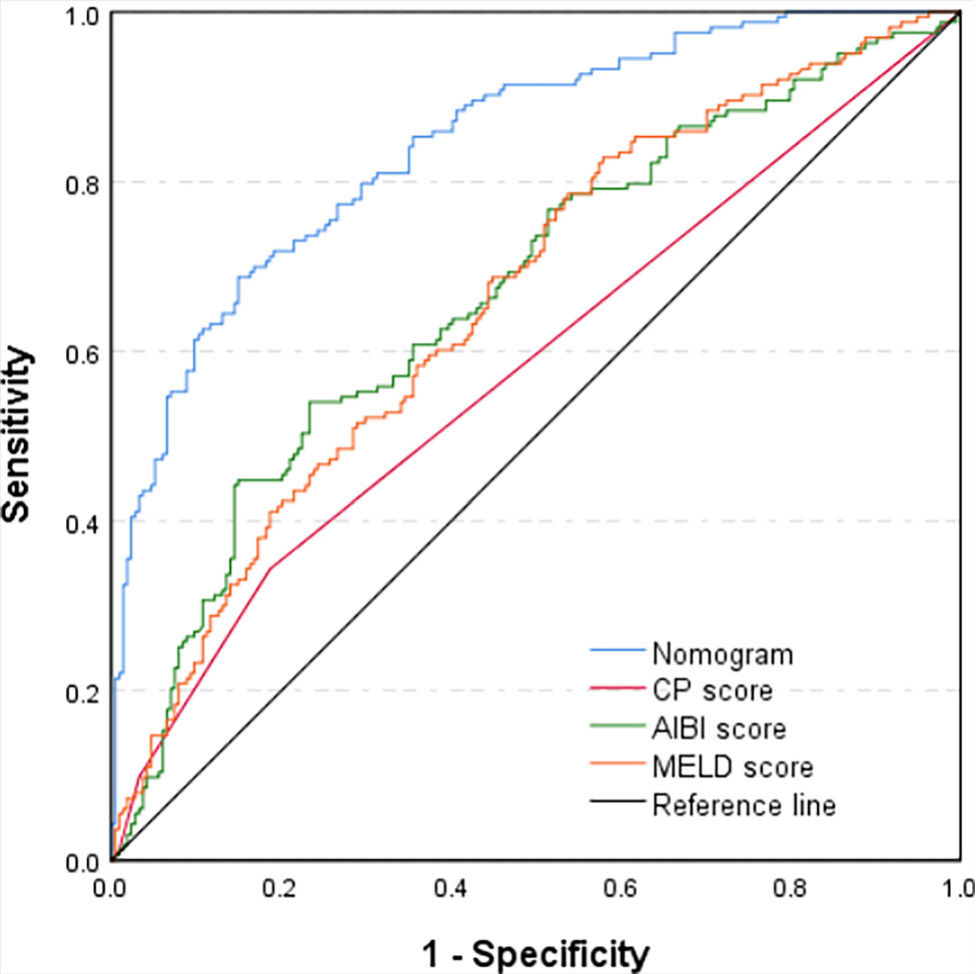
Comparison of predictive accuracy for post-hepatectomy liver failure between the nomogram and the conventional models (CP score, MELD score, and ALBI score) by the training cohort.

**Figure 3:**
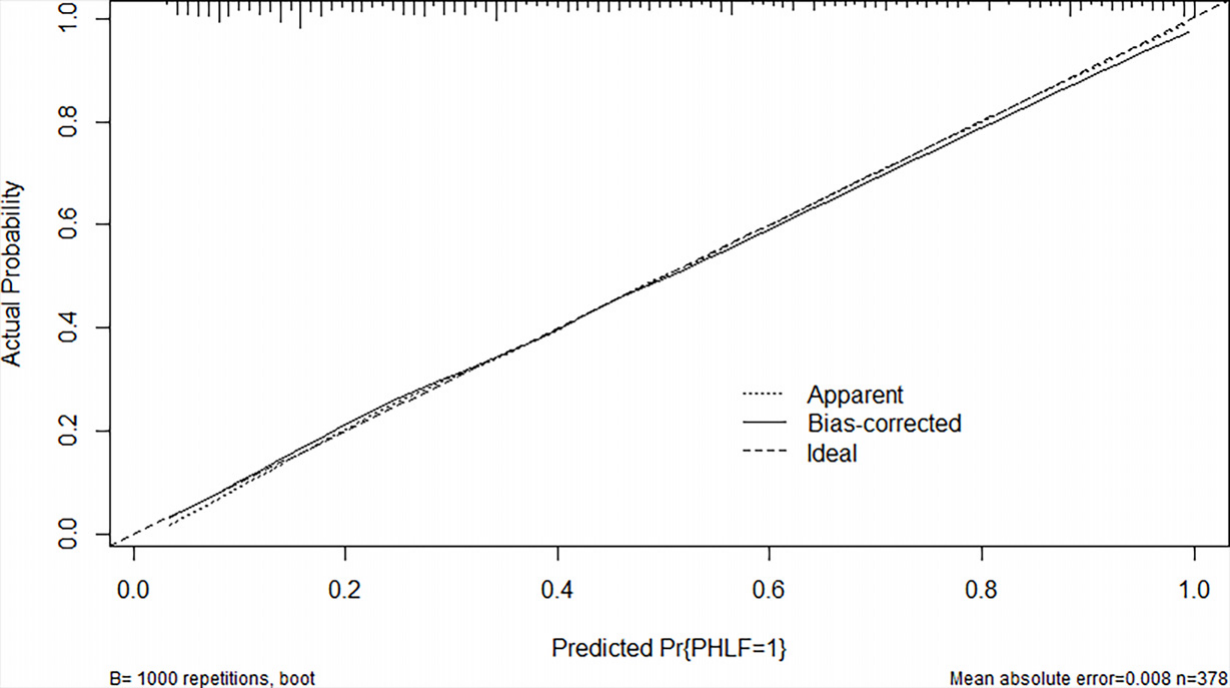
The calibration curve of the nomogram in the training cohort. The x-axis indicates the nomogram predicted probability of PHLF, and the y-axis represents the actual PHLF rate. The dotted line represents a perfect prediction, and the solid line represents the predictive performance of this nomogram. The closer the solid line fit is to the dotted line, the better the prediction of the nomogram will be.

Through multivariate analysis, we found that cirrhosis, PT, tumor size, ICG-R15%, blood loss and APRI were independent risk factors of PHLF. These independent risk factors were further integrated to establish a PHLF estimation nomogram in the training cohort (**Figure 1**). The nomogram showed a better accuracy for PHLF prediction, with a C-index of 0.845 (95%CI, 0.806-0.884) (**Figure 2**). The calibration curves for PHLF prediction revealed sufficient agreement between the nomogram and actual observation (**Figure 3**).

### Validation of the Nomogram

**Figure 4:**
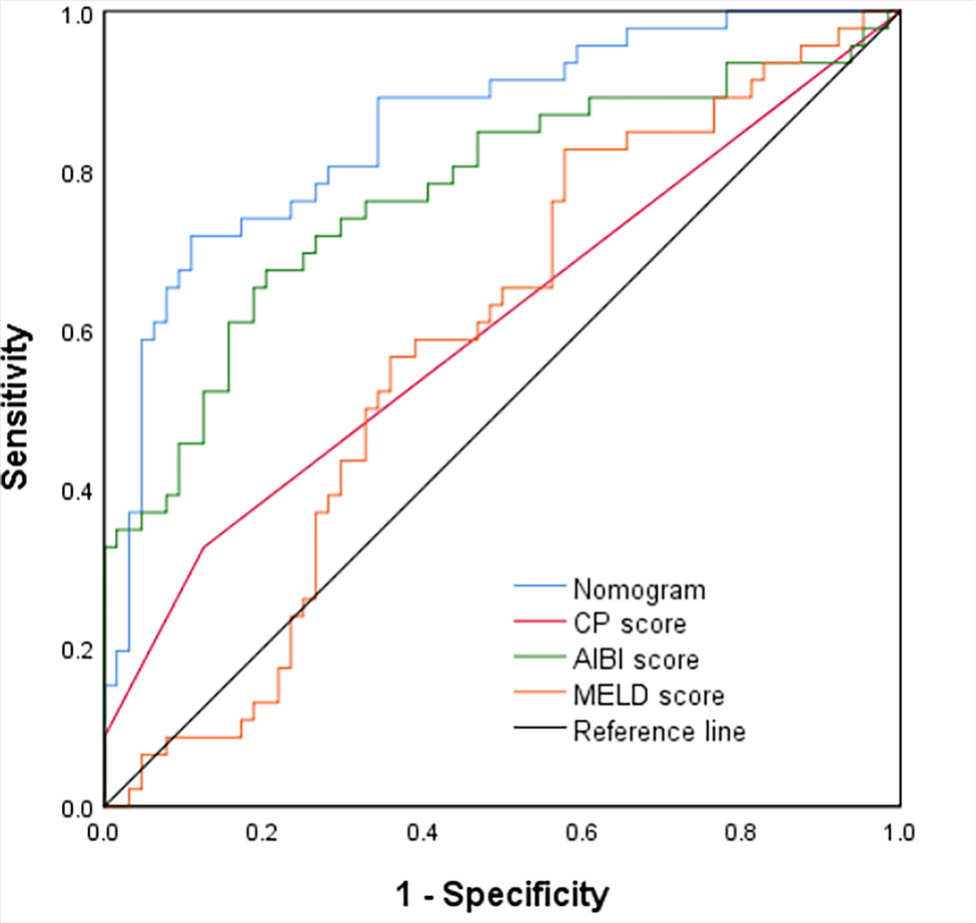
Comparison of predictive accuracy for post-hepatectomy liver failure between the nomogram and the conventional models (CP score, MELD score, and ALBI score) by the validation cohort.

**Figure 5:**
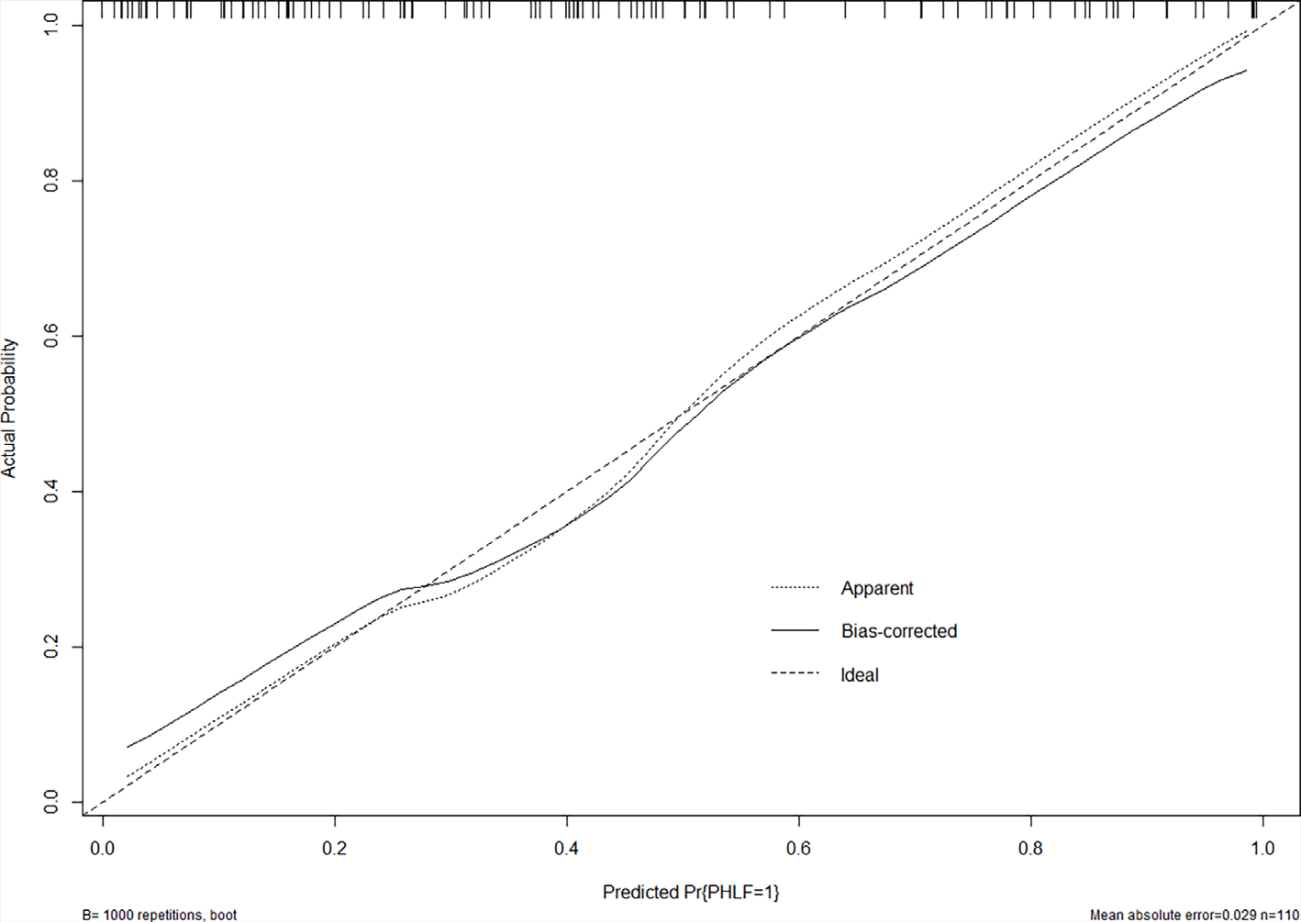
The calibration curve of the nomogram in the validation cohort. The x-axis indicates the nomogram predicted probability of PHLF, and the y-axis represents the actual PHLF rate. The dotted line represents a perfect prediction, and the solid line represents the predictive performance of this nomogram. The closer the solid line fit is to the dotted line, the better the prediction of the nomogram will be.

In the validation cohort, the nomogram also demonstrated a better accuracy for PHLF prediction, with a C-index of 0.854 (95%CI, 0.782-0.926) (**Figure 4**). The calibration curves for PHLF prediction showed good agreement between the nomogram and actual observation (**Figure 5**).

### Comparison of Predictive Accuracy for PHLF Between the Nomogram and the Conventional Models in the Training Cohort and Validation Cohort Respectively

**Figure 6:**
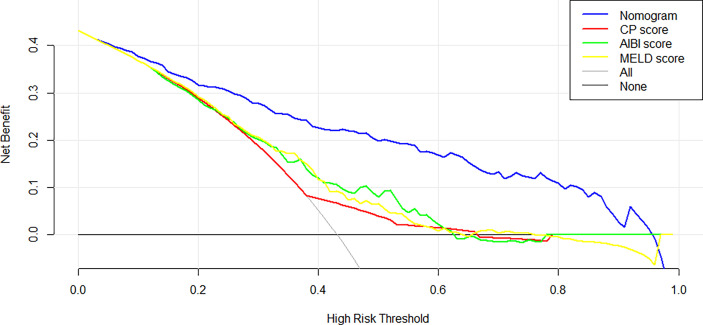
Decision curve analysis of nomogram and the conventional models in the training cohort.

**Figure 7:**
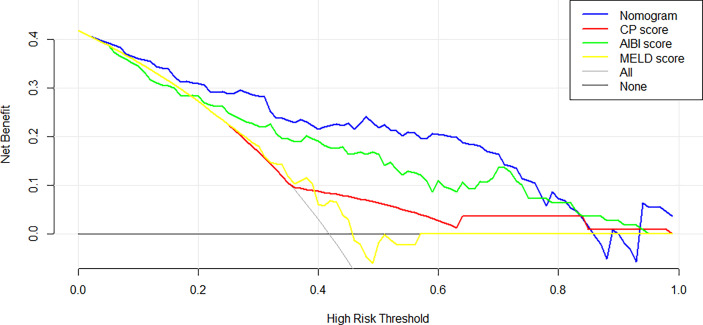
Decision curve analysis of nomogram and the conventional models in the validation cohort.

In the training cohort, the C-index of the nomogram was significantly higher than CP score (C-index=0.582, 95%CI, 0.523-0.640), ALBI score (C-index=0.670, 95%CI, 0.615-0.725), MELD score (C-index=0.661, 95%CI, 0.606-0.716) (**Figure 2**). DCA has been used to evaluate the clinical value of models that integrates the preferences of patients into the analysis (23, 24). DCA indicated that this nomogram of PHLF prediction added more benefit compared with CP score, ALBI score and MELD score (**Figure 6**). In the validation cohort, we can draw the same conclusion. The C-index of the nomogram was higher than CP score (C-index=0.606, 95%CI, 0.496-0.716), ALBI score (C-index=0.771, 95%CI, 0.678-0.865), MELD score (C-index=0.583, 95%CI, 0.476-0.690) (**Figure 4**). DCA of validation cohort showed that this nomogram was more reliable compared with conventional models too (**Figure 7**).

## Discussion

Post-hepatectomy liver failure is one of the most feared complications after hepatectomy in HCC patients. There is a need to prospectively identify HCC patients at risk of PHLF. Therefore, establishing a prediction model of PHLF is necessary to improve clinical decisions.

Many models have been put forward to predict the occurrence of PHLF. But the predictive model is still evolving due to its multifactorial causative factors (25). Based on our clinical data, we performed this study to recognize the risk of PHLF in HCC patients in order to construct a nomogram for predicting PHLF.

In our analysis, we found that tumor size, blood loss(≥400ml), cirrhosis, PT, ICG-R15 and APRI were the independent risk factors for PHLF in HCC patients through the multivariable logistic regression analysis. Based on the risk factors, we developed the nomogram to predict the occurrence of PHLF. As for tumor size, we think the size of tumor influence the scope of resection of liver parenchyma, consequently affecting the volume of the remaining healthy liver. Several reports suggested that patients with a smaller liver remnant have a greater chance of developing PHLF (26, 27). Also, Heng Zou and his team found liver remnant is a good predictor of PHLF (28). As for blood loss, Osamu Aramaki (29) in his article demonstrated that intraoperative blood loss was the most crucial factor related to postoperative complications, including PHLF. Also back in 2007, Marieke T. de Boer found that there is a significant and clinically relevant association between blood loss and postoperative mortality and morbidity (30). Considering that the liver has abundant blood flow, excessive bleeding inevitably leads to impairment of liver cells, with the liver function decline. Liver cirrhosis has a great effect on liver regeneration after hepatectomy. In other words, Cirrhosis is a negative predictor of liver regeneration and liver function. It is extremely important to evaluate the degree of cirrhosis since it is the dominant risk factor for both PHLF and the prognosis of HCC patients (31). HCC patients with severe cirrhosis have higher morbidity and mortality rates after hepatectomy when compared with non-cirrhotic patients (32). Our study also confirms this point of view that cirrhosis is an independent risk factor of PHLF. Prothrombin time (PT) is an important reflection of coagulation status. It represents an essential parameter in many models that evaluate liver function, such as the Child-Pugh score system. Similarly, PT plays an important role in PHLF according to several studies (33, 34). For decades, ICG-R15 has been applied to test liver function prior to hepatectomy. Especially in Eastern countries, ICG-R15 was the most common approach to select suitable HCC patients for liver resection (35, 36). Admittedly, tumor size, blood loss, cirrhosis, PT and ICG-R15 have been demonstrated to predict PHLF in many studies which are consistent with our conclusion.

In so many serum inflammatory indices, only APRI is a unique independent factor in predicting PHLF in our analysis. APRI was used to predict the degree of liver fibrosis in patients since it is a non-invasive test (37). In 2015, the World Health Organization recommend APRI for non-invasive evaluation for liver cirrhosis in patients with chronic hepatitis B infection. APRI consists of two components, AST and Plt. The progression of liver cirrhosis in HCC patients is inevitably accompanied by sustained damage to liver cells, which results in the release of AST and the increase of its concentration in peripheral blood (38). The platelet count could be decreased because of sequestration and destruction of platelets in the enlarging spleen (portal hypertension) (39). And, Thrombopoietin (TPO) synthesis in the liver is reduced because of liver cirrhosis which could stimulate platelet formation (40). These may explain why APRI, not other inflammatory indices could be used to predict PHLF. In our study, APRI has an OR value of 4.652, which is higher than the other independent risk factors. That means it has a higher correlation with PHLF than others. APRI presented in nomogram confirmed the conclusion as well.

Compared to CP score, ALBI score and MELD score, our nomogram performed well in predicting PHLF and its prediction was supported by the C-index (0.845 and 0.854 for the training cohort and validation cohort, respectively). Traditionally, nomogram is assessed using metrics of diagnostic performances such as specificity, sensitivity and the C-index which fail to determine the clinical value. DCA is a wide-used tool for assessing the benefit of a diagnostic test across a variety of patient preferences for recognizing risks of undertreatment and overtreatment to facilitate decisions about test selection and use. In our study, the DCA indicated that our nomogram brought more benefits than other models in the training cohort and validation cohort. So our nomogram could be used uniformly in clinical practice.

Our nomogram is helpful in predicting PHLF, which can guide therapeutic decisions. By doing this, specific monitoring strategies can be established according to the specific risk categories. For example, if HCC patients are evaluated as a high-risk group of PHLF, we would recommend early use of hepatic protectant, close supervision and intensive care after surgery.

To our knowledge, this is the first nomogram based on inflammatory indices and ICG-R15 to predict PHLF. We emphasized the importance of APRI in the prediction model. However, there are several limitations in the present study. The main limitation is its retrospective nature. Although our data came from two academic centers, our study has a relatively small sample size. A future multicenter study including a larger number of HCC patients is needed to confirm our findings. Then, as mentioned above, there are many definitions of PHLF, resulting in a wide variation in the incidence of PHLF. We could compare different diagnostic criteria to determine which one is more beneficial to patients. Finally, the main etiology of HCC was chiefly HBV. Even though it didn’t play an important role in the prediction of PHLF, it is necessary to include more populations with different etiologies such as alcoholic liver disease or HCV.

In conclusion, we demonstrated that tumor size, blood loss, cirrhosis, PT, ICG-R15 and APRI are the independent risk factors of prediction of PHLF. We present a novel prediction nomogram of PHLF by combining the independent risk factors. The nomogram showed a good predictive performance and would be a convenient tool for us to facilitate clinical decisions.

## Data Availability Statement

The original contributions presented in the study are included in the article/supplementary material. Further inquiries can be directed to the corresponding author.

## Ethics Statement

The study was approved by the Ethics Committee of the Xiangya Hospital of Central South University and the Second Xiangya Hospital of Central South University in compliance with the Declaration of Helsinki. Written informed consent was obtained from all patients for use of their data in this study.

## Author Contributions

TF and LeZ contributed to conception and design of the sstudy. TF and LeZ organized the database. TF and LeZ performed the statistical analysis. TF wrote the first draft of the manuscript. TF wrote sections of the manuscript. All authors contributed to the article and approved the submitted version.

## Funding

This study was funded by the National Nature Science Foundation of China (NO. 81771932).

## Conflict of Interest

The authors declare that the research was conducted in the absence of any commercial or financial relationships that could be construed as a potential conflict of interest.
